# Validation of an automated ELISA system for detection of antibodies to Aleutian mink disease virus using blood samples collected in filter paper strips

**DOI:** 10.1186/1743-422X-11-141

**Published:** 2014-08-08

**Authors:** Anna Knuuttila, Pirjo Aronen, Majvor Eerola, Ian A Gardner, Anna-Maija K Virtala, Olli Vapalahti

**Affiliations:** Department of Veterinary Biosciences, Faculty of Veterinary Medicine, University of Helsinki, Helsinki, Finland; Department of Virology, Haartman Institute, Faculty of Medicine, University of Helsinki, Helsinki, Finland; Fin Furlab Oy, Finnish Fur Breeders’ Association, Vaasa, Finland; Department of Health Management, University of Prince Edward Island, Charlottetown, PE Canada

**Keywords:** Aleutian mink disease virus, Bayesian analysis, Carnivore amdoparvovirus 1, Counter-current immunoelectrophoresis, Enzyme-linked immunosorbent assay, Sensitivity, Specificity

## Abstract

**Background:**

Aleutian mink disease virus (AMDV) is the cause of a chronic immune complex disease, Aleutian disease (AD), which is common in mink-producing countries. In 2005, implementation of an AMDV eradication programme in Finland created a need for an automated high-throughput assay. The aim of this study was to validate an AMDV-VP2 -recombinant antigen ELISA, which we developed earlier, in an automated assay format for the detection of anti-AMDV antibodies in mink blood and to determine the accuracy of this test compared with the reference standard (counter-current immunoelectrophoresis, CIEP).

**Methods:**

A blood sampling method based on filter paper 12-strips (blood combs) and a device to introduce these strips to an ELISA plate for elution of the samples were developed. Blood and serum samples were collected from 761 mink from two farms with low (2%) and high (81%) seroprevalences of AMDV infection in 2008. ELISA sensitivity and specificity were estimated with a Bayesian 2-test 2-population model that allowed for conditional dependence between CIEP and ELISA. Agreement between the two tests was assessed with kappa statistic and proportion agreement.

**Results:**

The sensitivity and specificity of the automated ELISA system were estimated to be 96.2% and 98.4%, respectively. Agreement between CIEP and ELISA was high, with a kappa value of 0.976 and overall proportion agreement of 98.8%.

**Conclusions:**

The automated ELISA system combined with blood comb sampling is an accurate test format for the detection of anti-AMDV antibodies in mink blood and offers several advantages, including improved blood sampling and data handling, fast sample throughput time, and reductions in costs and labour inputs.

## Background

Aleutian disease (AD), a common and economically significant disease in farmed mink (*Neovison vison*), is caused by Aleutian mink disease virus (AMDV). The virus belongs to the recently renamed species of *Carnivore amdoparvovirus 1* of the family *Parvoviridae* [1]. AMDV has a single-stranded DNA genome and encodes three non-structural (NS1, NS2, and NS3) and two structural proteins (VP1 and VP2) [2, 3]. The clinical presentation of AMDV infection varies from a non-progressive and non-persistent disease to a progressive, persistent, and potentially fatal immune complex disease (so-called classical AD) [2]. Susceptibility to disease persistence and progression depends on host and viral factors, and clinical disease is more severe in Aleutian genotype mink [2]. Infected adult mink typically develop high concentrations of anti-AMDV antibodies, which can be detected with counter-current immunoelectrophoresis (CIEP) from 5 days to 7 weeks post-infection [2, 4].

In 2005, the Finnish Fur Breeders’ Association (FFBA) implemented an eradication programme to depopulate AMDV-infected farms and reduce the overall prevalence of AMDV, and consequently, to improve the health status of animals and reduce economic losses to farmers caused by AD. Control and eradication of AMDV on farms currently depend on serological screening and culling of anti-AMDV antibody-positive animals, strict sanitary measures, disinfection of cages and equipment, and introduction of replacement animals from low-risk farms. In the programme, farms are classified into 5 groups, A-E, according to AMDV seroprevalence. In group A, test prevalence must be zero. In groups B, C, and D, within-farm prevalence should not exceed 1, 2, and 50 positive/1000 breeding females, respectively. In group E, prevalence exceeds 50 positive/1000 breeding females. Fin Furlab (formerly Fur Animal Feed Laboratory) tests 500,000 to 600,000 samples annually for anti-AMDV antibodies. From 2000 to 2012, the annual mean seroprevalence of all tested animals ranged between 3.3% and 13.6%.

A need for an automated replacement test for the traditionally used CIEP method arose due to implementation of an eradication programme, which led to increased numbers of samples, demand for an additional labour force, and problems in the availability of the CIEP antigen. The aim of this study was to automate and validate an enzyme-linked immunosorbent assay (ELISA) for detecting anti-AMDV antibodies in blood of farmed mink. Additional objectives were to compare the sensitivities (Se) and specificities (Sp) of ELISA and CIEP, develop a simple blood sampling method for ELISA, improve the sample throughput time, and decrease the labour intensity and costs of testing. Results of this study are reported according to standards for the reporting of diagnostic accuracy studies (STARD) statement [5], with some modifications for livestock studies [6].

## Results

### Animals and herds

**Figure 1:**
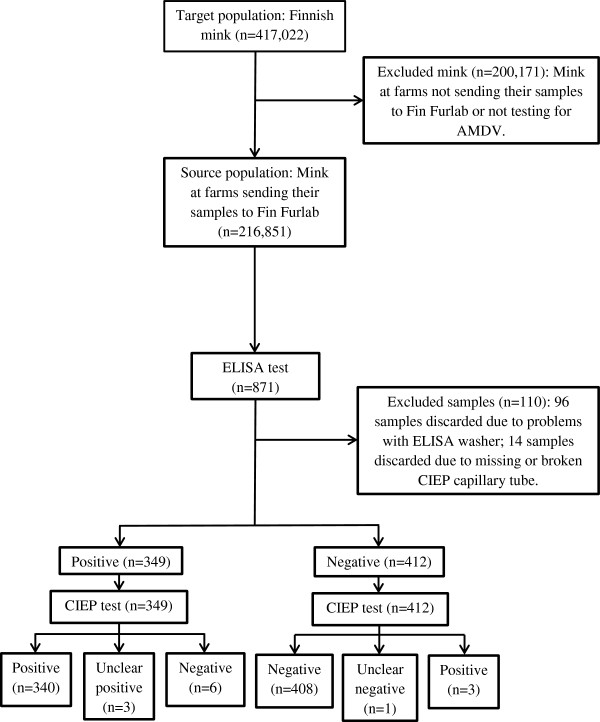
**Flow diagram of the sampling and number of mink undergoing CIEP and automated AMDV-VP2 ELISA tests.**

We sampled and screened for anti-AMDV antibodies on two mink farms and used both CIEP with capillary blood samples, and the automated ELISA system with recombinant VP2 antigen and filter-paper (FP) strips (designated blood combs below). Farm 1 (high AMDV seroprevalence) was situated in Kannus, Central Ostrobothnia, and had 1412 breeding mink, mainly brown, with a small proportion of other colour types. AMDV seroprevalence on this farm was 81% (in 2007). The farm had a history of clinical AD, including high mortality and low-quality fur. A total of 361 mink (25.6%) were sampled on January 9–17, 2008. All sampled mink were brown, aged 1 to 4 years (median 1 year); 1 was male and 360 were females. Farm 2 (low AMDV seroprevalence) was situated in Kaustinen, Central Ostrobothnia, and had 1367 breeding mink, mainly standard dark, Aleutian, and other colour types. AMDV seroprevalence was 2% (in 2007), and the farm had no history of clinical AD. Samples from 400 mink (29.3%) from farm 2 were taken on January 8–14, 2008; this included 319 black, 39 blackcross, and 42 brown mink. Their ages ranged from 1 to 4 years (median 2 years). Forty-five were males and 355 were females. The flow diagram of the sampling is shown in Figure 1.

### Indeterminate and missing results

Results of two ELISA plates (containing altogether 96 samples) were discarded due to problems with the washer. Fourteen CIEP results were missed as the capillary tube was lost or broken during sampling or shipping. If either the ELISA or CIEP sample could not be analysed, the animal was excluded from the analysis. CIEP results of 3 samples were regarded as unclear positive and 1 as unclear negative. These samples were included in the analysis. The ELISA optical density (OD) values for the unclear CIEP positive samples were 0.354, 0.616, and 1.056. Thus, the first was low also in ELISA. The OD value for the unclear CIEP negative sample was 0.116.

### ELISA and CIEP test results and Bayesian estimates of sensitivity and specificity

**Figure 2:**
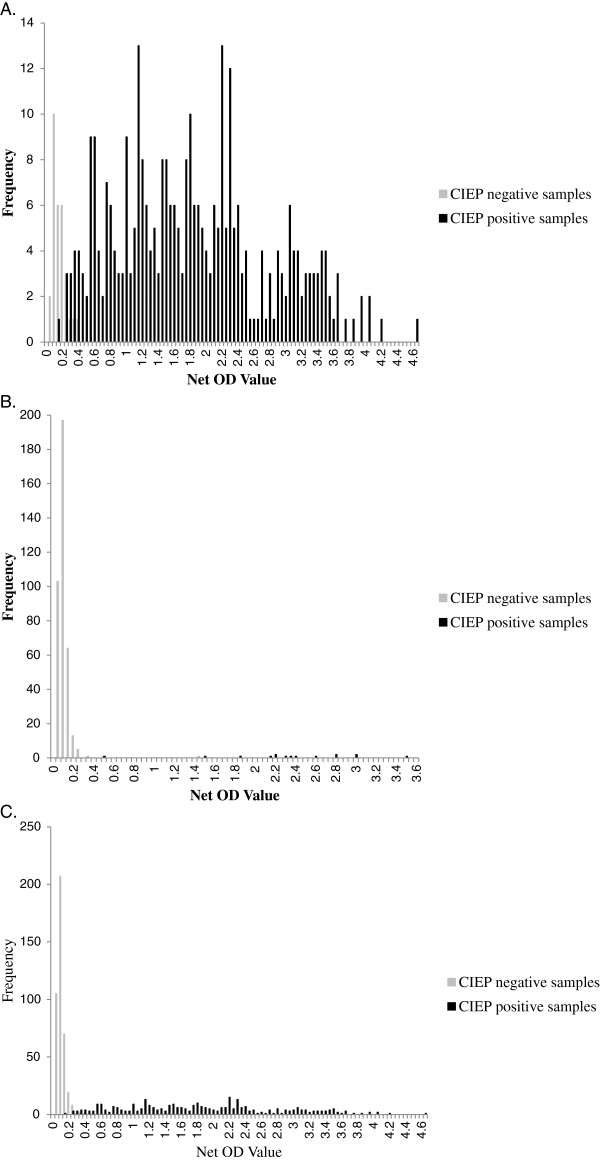
**Histograms of automated AMDV-VP2 ELISA results of CIEP-positive and -negative samples from mink. A**. Farm 1 (high prevalence), 361 samples. **B**. Farm 2 (low prevalence), 400 samples. **C**. All 761 samples. X-axis shows the categories (at 0.05 intervals) of net ELISA optical density (OD) values. Y-axis depicts the number of samples within each category.

**Figure 3:**
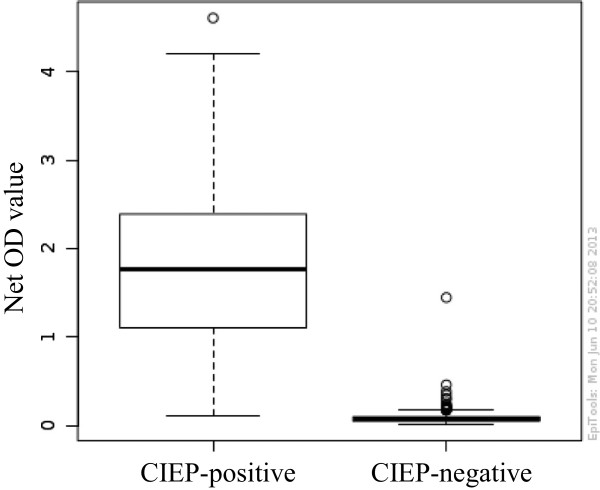
**Box-and-whisker plot graph of AMDV-VP2 ELISA results for CIEP-positive and -negative samples from mink.** The bottom of the box shows the 25^th^ percentile, the line within the box indicates the median, and the top of the box shows the 75^th^ percentile. Ends of the whiskers are at 25^th^ percentile – (1.5 × interquartile range) and 75^th^ percentile + (1.5 × interquartile range). Outliers are indicated as small circles. OD = optical density.

Comparisons of the positive and negative results of CIEP and the automated ELISA system for both farms are shown in Tables 1 and 2. ELISA results had a mean OD of 0.086 (range 0.013–1.447) for CIEP-negative and 1.826 (0.115–4.604) for CIEP-positive samples. Overall, there were nine discrepant results. Three CIEP-positive samples were negative in ELISA (OD ranging from 0.115 to 0.226) and 6 CIEP-negative samples were ELISA-positive (OD 0.299–1.447). Most values of discrepant samples were around the cut-off (0.115–0.461), with only 1 CIEP negative with high ELISA OD (1.447). Histograms of the distribution of the ELISA test values of farm 1, farm 2, and total are depicted in Figure 2A–C. The box-and-whisker plot graph is shown in Figure 3.

**Table 1:** **Cross-classified results for CIEP and automated AMDV-VP2 ELISA at farm 1 (high prevalence)**

		CIEP		
		Positive	Negative	Total
**ELISA**	**Positive**	328	3	331
	**Negative**	3	27	30
	**Total**	331	30	361

**Table 2:** **Cross-classified results for CIEP and automated AMDV-VP2 ELISA at farm 2 (low prevalence)**

		CIEP		
		Positive	Negative	Total
**ELISA**	**Positive**	15	3	18
	**Negative**	0	382	382
	**Total**	15	385	400

**Figure 4:**
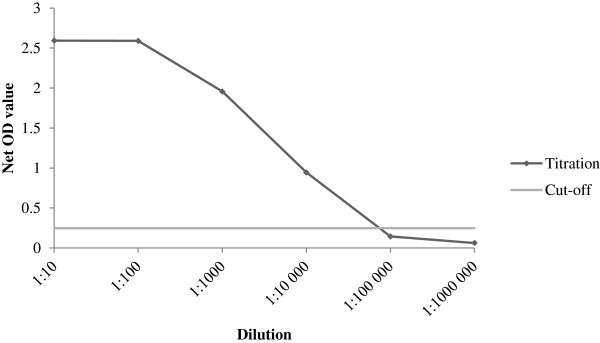
**End-point titration curve of automated AMDV-VP2 ELISA using a high-positive mink serum.** The limit of detection of the positive serum was 1:10,000 in ELISA and 1:100 in CIEP. OD = optical density.

The limit of detection of positive serum was 1:10,000 in ELISA (Figure 4) and 1:100 in CIEP. No cross-reactivity against mink enteritis virus, *Pseudomonas aeruginosa*, and *Clostridium botulinum* was evident in the ELISA results.

The tests showed almost perfect agreement [7], with a kappa value of 0.976 (95% confidence interval (CI); 0.961–0.992), overall proportion agreement of 98.8% (95% CI; 97.9–99.4%), proportion positive agreement of 98.7% (95% CI; 97.7–99.8%), and proportion negative agreement of 98.9% (95% CI; 97.0–99.4%).

The median Se and Sp of ELISA from the Bayesian model using informative priors on CIEP test performance and prevalence (model 2) were 96.2% and 98.4%, respectively (Table 3). The probability that ELISA Se (Sp) was greater than the respective parameters for CIEP was 58% (55%), indicating comparable accuracy in these 2 populations. A sensitivity analysis using informative priors on a single parameter only (models 1 and 3) produced changes in Se estimates of about 3% in both tests, but virtually no change in Sp. The performance characteristics of ELISA and CIEP were very similar, with few discordant results. In the high prevalence population, there were only 6 discordant results (Table 1) and the discordance was symmetric, which meant that the sensitivities were essentially identical. In the low prevalence population, the discordance was lower and asymmetric (3 vs 0) (Table 2), resulting in similar specificities.

**Table 3:** **Results of the 2-test 2-population Bayesian modelling estimating AMDV-VP2 ELISA sensitivity and specificity**

Model		Median	95% probability interval
**1. CIEP sensitivity/specificity only**	**ELISA sensitivity**	0.945	0.898–0.987
	**ELISA specificity**	0.984	0.953–0.998
	**CIEP sensitivity**	0.945	0.898–0.987
	**CIEP specificity**	0.978	0.945–0.995
	**Prevalence (high)**	0.964	0.912–0.998
	**Prevalence (low)**	0.027	0.002–0.056
**2. CIEP sensitivity/specificity and prevalences**	**ELISA sensitivity**	0.962	0.915–0.990
	**ELISA specificity**	0.984	0.953–0.998
	**CIEP sensitivity**	0.961	0.915–0.989
	**CIEP specificity**	0.977	0.945–0.995
	**Prevalence (high)**	0.943	0.903–0.984
	**Prevalence (low)**	0.026	0.003–0.054
**3. Prevalences only**	**ELISA sensitivity**	0.977	0.930–0.994
	**ELISA specificity**	0.985	0.954–0.999
	**CIEP sensitivity**	0.979	0.931–0.995
	**CIEP specificity**	0.979	0.946–0.996
	**Prevalence (high)**	0.931	0.895–0.974
	**Prevalence (low)**	0.027	0.003–0.054

Conditional correlations between ELISA and CIEP results were always positive in models 1–3 (data not shown), providing statistical evidence (in addition to the biological argument) that the dependence model was more appropriate than a conditional independence model.

### Prevalence and predictive values based on ELISA results

In all models, the median true seroprevalence of AMDV infection in the high prevalence population was estimated to be between 93% and 96%, and approximately 3% in the low prevalence population. Prevalence in the high prevalence population was lower in models 2 to 3, with a concomitant increase in test Se (Table 3). The absolute difference (3%) was considered acceptable, and conclusions about comparative test accuracy did not change. For the high prevalence (94.3%) population, the median positive predictive value (PPV) was 99.9% (95% probability interval (PI); 99.7–99.99%) and negative predictive value (NPV) 61.2% (95% PI; 17.8–90.0%). For the low prevalence (2.6%) population, the median PPV was 62.1% (95% PI; 6.5–96.0%) and NPV 99.9% (95% PI; 99.6–99.99%).

### Test repeatability

Within-run variability, between-run variability, and between-serial repeatability for the negative serum measured as coefficient of variation (CV) were 4% (OD 0.100 ± standard deviation (SD) 0.004), 9% (0.107 ± 0.010), and 8% (0.102 ± 0.008), respectively, and for the low-positive serum 8% (0.425 ± 0.032), 26% (0.532 ± 0.139), and 6% (0.746 ± 0.043), respectively.

## Discussion

In Finland, the CIEP test has been used for screening anti-AMDV antibodies since 1980. In 2005, the FFBA implemented a new eradication programme to decrease AMDV prevalence and help farmers to eradicate the virus from mink farms. From this arose a need to develop a new, automated, efficient, and highly sensitive and specific test method. In eradication programmes, a high Se is needed for screening in order to identify potent animals and prevent the spread of the virus. However, in the absence of a confirmatory test, a high Sp is also required to achieve a high PPV at low prevalence, to avoid unnecessary culling and reduce economic losses (culling valuable breeding animals, loss of group A status, and effects on animal trade) to farmers caused by false-positive results.

The CIEP, developed in the 1970s [8], is used for routine screening of anti-AMDV antibodies in many countries. Estimated Se and Sp for CIEP vary from 78.7% to 98.9% and from 90.4% to 100%, respectively [9–12].

Few studies on use of ELISA tests in anti-AMDV antibody screening have been reported [9, 12–14]. Wright and Wilkie [9] used a fluorocarbon-activated antigen and stated that due to the high number of false-negative results ELISA was not as effective as CIEP as a screening method. Dam-Tuxen et al. [12] reported results of an automated ELISA based on cell-culture derived AMDV-G antigen (ELISA Danad antigen, Kopenhagen Fur, Glostrup, Denmark) and dried blood spot cards and concluded that CIEP and ELISA have comparable performance. Andersson et al. [13] compared Se and Sp of two manual ELISA tests to CIEP. One of the ELISAs was based on the same recombinant AMDV-VP2 antigen used here and the other was an ELISA kit based on a traditionally cultured AMDV-G antigen (Scintilla Development Company LLC, Bath, PA, USA). The recombinant VP2 ELISA had a high Se, in contrast to the AMDV-G ELISA kit. Our previous study [14] focused on development of the recombinant antigen, showing its antigenicity and optimizing the ELISA method for diagnostic purposes with a preliminary comparison of CIEP and ELISA results.

Our findings here on the Se (96.2%) and Sp (98.4%) of the automated ELISA system were in concordance with these earlier results [13, 14]. The Se and Sp of ELISA and CIEP did not differ substantially regardless of the selected model and were similar for both assays. Nine discordant results were detected between ELISA and CIEP (Tables 1 and 2). These could be caused by differences in the antibody detection level, differences in the ability to identify antibodies against different AMDV strains, cross-reactions, and non-specific precipitin lines in CIEP. Also, differences in antigen composition (CIEP uses a whole virus antigen and ELISA a recombinant VP2 antigen) and antigen cultivation and production techniques could cause some discrepancies in the results.

Overall, the repeatability of the ELISA was high, with a CV under 10%, except for the low-positive serum with a between-run CV of 26%. The higher variability for a low-positive sample was expected, as a sample at a level between the cut-off and high-positive value will express variability more frequently than negative and high-positive samples. As none of the low-positive values were below the cut-off value and the assay is used qualitatively instead of quantitatively, it was not considered to be a major problem. Furthermore, in the future this issue can be controlled by normalizing the data, for instance, by expressing the results as sample-to-positive control ratio (S/P-ratio).

The probability that a test-positive animal is actually infected was only 62.1% on the low prevalence farm. This problem can be minimized by using additional confirmatory tests for positive reactors (serial testing) in AMDV-free and low prevalence farms to increase the Sp and PPV.

**Figure 5:**
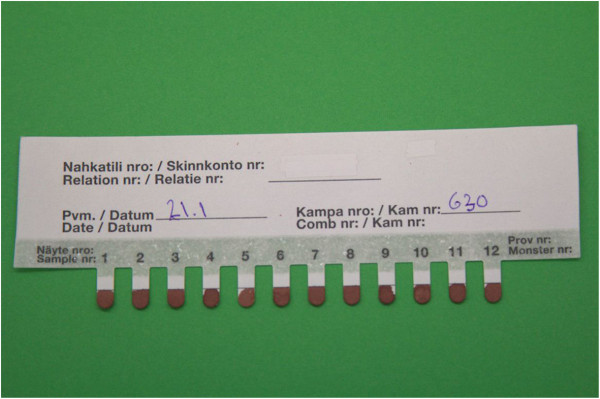
**Photograph of the filter paper blood comb.** The blood comb is used for the blood sampling of mink in the automated AMDV-VP2 ELISA system. Twelve mink can be sampled with one comb. Except for the tips, the combs are coated with a thin film to prevent the comb absorbing dilution buffer. On the upper part of the comb, there is room for farm identification, date, and serial number.

**Figure 6:**
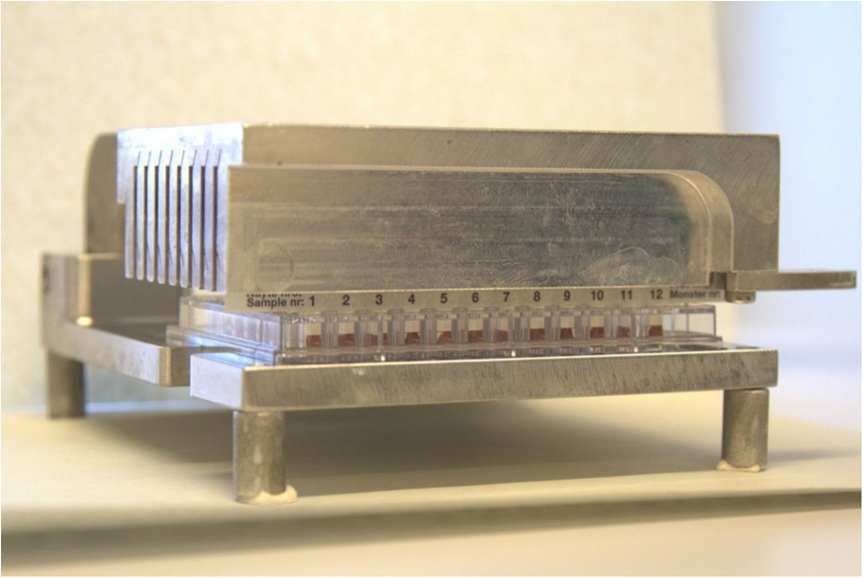
**Photograph of the device utilized in the automated AMDV-VP2 ELISA.** The device introduces eight blood combs simultaneously to an ELISA plate for the elution of antibodies.

To fully utilize the automated system and to avoid the time-consuming and laborious introduction of serum from glass capillary tubes to the ELISA plate, a new blood sampling method, a FP 12-strip (so-called blood comb) was developed (Figure 5). FP blood sampling was first introduced by Guthrie and Susi in the 1960s [15] and has since then been widely used in human medicine [16]. Various applications also exist in veterinary medicine [17]. However, in earlier ELISA applications an additional elution/dilution step is often needed. The blood comb described here can be directly introduced to an ELISA plate for elution of antibodies to the ELISA buffer without a separate elution/dilution step. The blood comb has several other benefits. The amount of blood needed is small, sampling is quick and easy, the samples do not get mixed up, go missing or get broken, their coding, shipment, and storage is easy, and a centrifugation step is not needed, unlike for capillaries. Moreover, to speed up the sample handling and to prevent variation caused by differences in the elution and incubation times of the samples, a device that introduces the blood combs to the ELISA plate was developed (Figure 6). This new blood sampling method also enables the pooling of several samples (by placing several combs into one row of wells), especially from AMDV-free farms, lowering the costs of testing.

The introduction of the automated ELISA for the screening of anti-AMDV antibodies in the current testing laboratory in Finland (Fin Furlab) in 2008 has increased testing capacity and decreased sample throughput time for large batches. Although new investments had to be made, the savings, especially from labour costs, have outweighed the costs. Previously, CIEP results were read visually and data were stored in a paper format. In the automated ELISA system, the robot software calculates and presents the positive and negative results in a spreadsheet format and stores them for further analysis. Another advantage is the unrestricted availability of the (in-house) recombinant VP2 antigen [14].

## Conclusions

Our aim was to automate and validate an ELISA system for detecting anti-AMDV antibodies in the blood of farmed mink. This automated ELISA system based on Finnish recombinant VP2 antigen and blood comb sampling was compared with manual CIEP with traditionally cultivated (Danish Danad) antigen and glass capillary sampling. The automated ELISA system described in this study is a highly sensitive and specific test for the serodiagnosis of AMDV infection, and it offers several advantages, including improved and simpler blood sampling and data handling, fast sample throughput time, and reductions in costs and labour intensity. The automated ELISA system has potential utility for demonstration of freedom from AMDV infection on mink farms or in individual animals, for facilitating AMDV eradication on infected farms, and for estimation of within-farm seroprevalence of AMDV.

## Methods

### Animals and herds

#### Study sampling frame

Mink farming in Finland is concentrated in the regions of Ostrobothnia, North, South, and Central Ostrobothnia of western Finland, where 95% of mink farms are situated. The target population of this study was Finnish farmed mink and the source population was mink from farms sending their anti-AMDV antibody screening samples to Fin Furlab. The study sample comprised a convenience sample from mink on farms willing to cooperate in the study. The seroprevalence and distribution of AMDV in the target population were unknown, as all farms do not test (52% of farms tested in 2007), but are suspected to be higher than in the source population (7.2% in 2007), as approximately 54% of tested farms were in group A that year. A flow diagram of the sampling is shown in Figure 1.

#### Selection of animals and herds

Two farms were selected among eligible farms meeting the inclusion criteria: farms with either low or high AMDV seroprevalence, breeding mink representing different genotypes and having different clinical presentations of AD (from non-progressive and non-persistent to progressive and persistent) in the year before sampling, and mink vaccinated against mink enteritis virus, haemorrhagic pneumonia caused by *Pseudomonas aeruginosa*, and botulism.

#### Sampling protocol and study design

Blood samples were collected during a routine screening for anti-AMDV antibodies by toenail cutting by a professional sample collector (Tarja Hinkkanen, FFBA), who took both the glass capillary tube (80 μl of blood) and blood comb (3 μl of blood is expected to saturate each comb) samples (Figure 5). Both samples were taken at the same time from each animal. Blood comb samples were air-dried prior to transportation. Samples were transported at ambient temperature to Fin Furlab the same day, the capillaries were centrifuged, and both samples were stored at +4°C. The following day, the blood combs were analysed with ELISA and the sera from the glass capillaries with CIEP.

Sample collection was planned before the ELISA and CIEP tests were performed (prospective study), and cross-sectional sampling with complete verification with the reference test was done.

### Test methods

#### CIEP - the reference standard

Although an imperfect reference standard, the CIEP test was selected because it was the only commercially-available test for large-scale diagnostics in mink globally and it has long been used for serological screening of AMDV. CIEP was performed using a commercial antigen and following the manufacturer’s instructions (Antigen Laboratory of the Research Foundation of the Danish Fur Breeders’ Association, Glostrup, Denmark). For a detailed description of the method, see Cho and Greenfield [18]. Results for CIEP were expressed as positive, negative, or unclear negative/positive. CIEP tests were performed and the results read by one co-author (ME) and a laboratory analyst (Pia Söderback), with 20 and 10 years of experience, respectively, with the assay.

#### The automated ELISA system

The ELISA method and production of recombinant VP2 antigen in insect cells have been previously described by our group [14]. This ELISA method was applied with some modifications: automation and robotics were used (see below), and blood samples were taken with a newly developed sampling method with a FP blood comb (see below and Figure 5). Blood combs were introduced to a 96-well ELISA plate containing 100 μl of dilution buffer, soaked for 2 min, after which the combs were discarded and the plate was incubated at room temperature for 1 h. A single sample was collected and measured from each animal, and results were calculated as net OD values (mean OD for two blank wells was subtracted from the raw OD value). Each plate contained duplicates of negative and positive controls and blank wells (containing all reagents except blood). The negative control, a serum of a known AMDV-negative (tested with Western blot, CIEP, and manual ELISA) mink, was diluted to 1:4 in dilution buffer. The positive control, a serum of an AMDV-positive (tested with PCR, CIEP, and manual ELISA) mink, was diluted to 1:300. Three microlitres of the diluted control serums were pipetted to the control combs, air-dried, and used in the ELISA test the same day. Control sera were stored at −20°C in 500 μl (negative) and 100 μl (positive) aliquots, thawed prior to use, and subsequently stored at +4°C until finished. The ELISA cut-off (0.248) was set to a point that gave maximal values for both Se and Sp calculated with EpiTools [19] using receiver operating characteristics (ROC) curve analysis. ELISA tests were done by two co-authors (ME and PA) with 12 months of experience with the assay. The laboratory personnel were aware of source farm identity. The CIEP and ELISA tests were run simultaneously, thus, personnel were unaware of the results of individual animals. Histograms of the ELISA results were generated with Excel 2010 (Microsoft Corp., Redmond, WA, USA) and the box-and-whisker plot graph with EpiTools [19].

A Cat X robot, Wellwash AC washer, three Multidrop 384 dispensers, and Multiscan Ascent EX spectrophotometer were supplied by Thermo Labsystems (Vantaa, Finland). The following steps were automated: coating of plates with prediluted antigen, dispensing the dilution buffer for samples, washing, dispensing of prediluted conjugate, 3,3’,5,5’ –tetramethyl benzidine and stopping solution (0.5 M H_2_SO_4_), reading, raw data capture, and after implementation of the cut-off also categorizing, presenting, and storing negative and positive results. The automated ELISA system was programmed using the software Polara 2.3.

A new system, blood combs (Figure 5), for collecting the blood samples and introducing them to the ELISA plate was developed. The blood combs were made from filter paper (Tervakoski Oy, Tervakoski, Finland).

A device (Figure 6) that introduces 8 blood combs simultaneously was manufactured by CNC-Tekniikka Oy (Vaasa, Finland).

### Statistical methods

For estimation of an approximate sample size in a 2-test 2-population Bayesian model, we first assumed that each population would have 100% or 0% AMDV infection, that a conservative estimate of ELISA Se and Sp would be 90% (estimates from [13] and [14] were at least 97% for both parameters), and that the estimate would have an error margin of 5% with 95% confidence. Based on a normal approximation to the binomial, the sample size for estimating Se and Sp was 138 in both the 100% and 0% populations. To account for test prevalence information from 2007 on the 2 study farms (81% and 2%), we targeted sample sizes of approximately 400 per population.

Analytical Se was estimated with end-point dilution of the positive control serum. The serum was tested in serial dilutions (2 replicates/dilution) in both ELISA and CIEP. Six 10-fold dilutions ranging from 1:10 to 1:10^6^ were used. The detection limit for the ELISA test was set to a dilution that yielded positive results on replicates (above the cut-off point). Analytical Sp against other pathogens present in mink was assessed from cross-reactivity against mink enteritis virus, *Pseudomonas aeruginosa*, and *Clostridium botulinum* by testing vaccinated animals. The ELISA test’s ability to identify antibodies against different AMDV strains circulating in Finland has been reported previously [14, 20].

Agreement between the two tests was assessed with EpiTools [19] by calculating kappa statistic and proportion agreements (overall, positive, and negative). Ninety-five percent confidence intervals for proportion agreement were calculated with EpiTools using Jeffreys interval [21].

Se and Sp were estimated with a 2-test 2-population Bayesian model that allowed for conditional dependence between CIEP and ELISA results, as described in Georgiadis et al. [22]. The model was run in WinBUGS 1.4.3 [23] using code described in Branscum et al. [24] and available elsewhere [25]. The 2-test 2-population dependence model requires informative prior data on two parameters to ensure identifiability. We used data on prevalence in the two populations from 2007 and the expert opinion of the first author based on previous studies about the Se and Sp of CIEP to derive informative beta priors (Table 4.) Non-informative (beta (1,1)) priors, which allowed all values between 0 and 1 to have equal probability, were used for other model parameters. After discarding the first 5000 samples as burn-in, we used the next 50,000 iterates for inferences about Se, Sp, and prevalence. We used the STEP function in WinBUGS to estimate the probability that the Se (Sp) of ELISA was greater than the Se (Sp) of CIEP. Model convergence was assessed by visual inspection of trace plots and running three chains from dispersed initial values. Predictive values and 95% PI were calculated for the high and low prevalence populations using the model with informative priors on CIEP accuracy and prevalences.

**Table 4:** **Informative beta priors used in the estimation of AMDV-VP2 ELISA sensitivity (Se) and specificity (Sp) with a 2-test 2-population Bayesian model**

Parameters		Most probable value	95% sure that at least	95% sure that less than	Beta prior
**Prevalence**	**Farm 1**	80%	50%		7.55, 2.64
	**Farm 2**	2%		20%	1.30, 15.80
**ELISA**	**Se**	99%	50%		4.40, 1.03
	**Sp**	97%	50%		4.58, 1.11
**CIE**	**Se**	80%	50%		7.55, 2.64
	**Sp**	95%	50%		4.77, 1.20

Within-run, between-run, and within-serial repeatability variability was assessed by using 1 negative, 1 low-positive mink serum, and 3 different batches of antigen. Negative and positive sera were diluted and handled as described above for the control sera. Within-run variability was calculated for each serum from 4 replicates on a single plate. Duplicates of each serum were analysed from 20 runs on 5 different days to assess the between-run variability. Within-serial repeatability was tested from duplicates of each serum against 3 different batches of antigen. The variability with ELISA quantitative outcome was estimated using CV% (SD ÷ mean × 100%). An evaluation of ELISA reproducibility was not performed because the Fin Furlab was the only laboratory using this ELISA test at the time of the study.

## Abbreviations

AD: Aleutian disease
AMDV: Aleutian mink disease virus
CI: Confidence interval
CIEP: Counter-current immunoelectrophoresis
CV: Coefficient of variation
ELISA: Enzyme-linked immunosorbent assay
FFBA: Finnish Fur Breeders’ Association
FP: Filter paper
NPV: Negative predictive value
OD: Optical density
PI: Probability interval
PPV: Positive predictive value
SD: Standard deviation
Se: Sensitivity
Sp: Specificity
STARD: Standards for the reporting of diagnostic accuracy studies
VP2: Structural protein 2.
